# A Comparative Meta-Analysis and *in silico* Analysis of Differentially Expressed Genes and Proteins in Canine and Human Bladder Cancer

**DOI:** 10.3389/fvets.2020.558978

**Published:** 2020-11-16

**Authors:** Victoria Vitti Gambim, Renee Laufer-Amorim, Ricardo Henrique Fonseca Alves, Valeria Grieco, Carlos Eduardo Fonseca-Alves

**Affiliations:** ^1^Department of Veterinary Surgery and Animal Reproduction, School of Veterinary Medicine and Animal Science, São Paulo State University—UNESP, Botucatu, Brazil; ^2^Department of Veterinary Clinic, School of Veterinary Medicine and Animal Science, São Paulo State University—UNESP, Botucatu, Brazil; ^3^John A. Paulson School of Engineering and Applied Sciences, Harvard University, Cambridge, MA, United States; ^4^Department of Veterinary Medicine, Università degli Studi di Milano, Milan, Italy; ^5^Institute of Health Sciences, Paulista University—UNIP, Bauru, Brazil

**Keywords:** transitional cell carcinoma, tyrosine kinase, gene ontology, dog, comparative oncology

## Abstract

Canine and human bladder cancer present similar anatomical, morphological, and molecular characteristics, and dogs can be considered a model for human bladder cancer. However, the veterinary literature lacks information regarding cross-validation analysis between human and canine large-scale data. Therefore, this research aimed to perform a meta-analysis of the canine literature on bladder cancer, identifying genes and proteins previously evaluated in these studies. In addition, we also performed a cross-validation of the canine transcriptome data and the human data from The Cancer Genome Atlas (TCGA) to identify potential markers for both species. The meta-analysis was performed using the following indexing terms: “bladder” AND “carcinoma” AND “dog” in different international databases, and 385 manuscripts were identified in our initial search. Then, several inclusion criteria were applied, and only 25 studies met these criteria. Among these studies, five presented transcriptome data, and 20 evaluated only isolated genes or proteins. Regarding the studies involving isolated protein analysis, the HER-2 protein was the most studied (3/20), followed by TAG-72 (2/20), COX-2 (2/20), survivin (2/20), and CK7 (2/20), and the remaining nine studies evaluated one isolated protein each. Regarding the cross-validation analysis of human and canine transcriptome data, we identified 35 dysregulated genes, including *ERBB2, TP53, EGFR*, and *E2F2*. Our results demonstrate that the canine literature on bladder cancer previously focused on the evaluation of isolated markers with no association with patient survival. This limitation may be related to the lack of a homogenous protocol for treating patients and the lack of follow-up during treatment. In addition, the lack of information regarding tumor muscle invasion can be considered an important limitation when comparing human and canine bladder tumors. Our *in silico* analysis involving canine and human transcriptome data provided several genes with the potential to be markers for both human and canine bladder tumors, and these genes should be considered for future studies on canine bladder cancer.

## Introduction

Transitional cell carcinoma (TCC), also called urothelial carcinoma, is the most common bladder cancer in both humans and dogs, which share clinical, pathological, and molecular alterations (1–3). In the United States, 81,400 new cases and 17,980 bladder cancer-related deaths are expected in 2020 (4). The last global cancer statistics (GLOBOCAN) estimated 549,393 new cases and 199,922 bladder cancer-related deaths in 2018 (5). In dogs, urothelial carcinoma is the most common malignant tumor in the canine bladder, representing 1% of all neoplasms that affect dogs (6). In humans, TCC is a tumor associated with several factors, such as cigarette smoking, occupational exposure (7), arsenic, cyclophosphamide, arylamines, and polycyclic aromatic hydrocarbons (8). In pet dogs, a case–control study was previously performed to correlate cigarette smoke, obesity, and use of topical insecticides and chemicals used at home with canine bladder cancer development (9). The authors found a high risk of bladder cancer development in obese dogs and dogs that used topical insecticides (9). Since dogs and humans share the same environment, dogs can be considered a model system for humans (10).

Canine and human TCCs are usually locally infiltrative cancers that can extend throughout the entire bladder, including the submucosa and muscular layers (11). Usually, human bladder carcinomas are superficial tumors (70% of cases) and are classified as non-muscle-invasive bladder carcinomas (NMIBCs) (11). Though NMIBC presents a good prognosis, muscle-invasive bladder carcinoma is considered a therapeutic challenge (11, 12). Thus, in the human literature, muscle-invasive bladder carcinoma has been the focus of recent studies. In dogs, determining the degree of infiltration is not standardized, since in several cases, tissue samples come from cystoscopy (1, 13). Since during cystoscopy a superficial small piece of tissue is collected from different areas, it is not usually possible to have tumor specimens containing deep layers, such as the muscular layers. In addition, human and canine bladder carcinoma can invade adjacent tissues and organs such as the ureter, prostatic urethra, and prostate gland (12).

The molecular phenotype of human bladder cancer is widely studied, and some genomic subtyping was previously proposed (14, 15). The Cancer Genome Atlas (TCGA) database revealed 64 significantly mutated genes in human TCCs responsible for different cellular processes, such as evasion of DNA repair and apoptosis and cell proliferation. In addition, there are subtypes of human muscle-invasive bladder cancer: luminal-papillary, luminal-infiltrated, luminal, basal-squamous, and neuronal subtypes (16). However, few papers perform molecular characterization of canine bladder cancer, though it is considered a promising area, and the molecular characterization of canine bladder carcinoma can provide valuable information regarding the biological behavior of this tumor (17).

In dogs, some recent studies performing transcriptome analysis revealed several important molecular findings, such as different differentiation degrees of canine bladder cancer in molecular subtypes categorized according to the *BRAFV595E* somatic mutation (*BRAFV600E* in humans) (18, 19), the identification of therapeutic targets (*PTGER2, ERBB2, CCND1, VEGF*, and *EGFR*), and categories based on basal and luminal subtypes, as for human bladder cancers, enabling the comparison of muscular invasion potential between dogs and humans (1). Therefore, studies performing molecular comparisons between human and canine bladder carcinomas can provide a unique opportunity to study this cancer subtype in both species. In this regard, this manuscript aimed to perform a literature meta-analysis and extract all information regarding gene and protein expression in canine bladder cancer and perform an *in silico* analysis to identify common gene alterations among dogs and humans to select candidates for future studies regarding prognosis or treatment.

## Materials and Methods

### Study Design

The study design is summarized in Supplementary Figure 1. We divided the study methods in three steps: (1) meta-analysis of the previous literature aiming to identify dysregulated genes and proteins in canine bladder cancer; (2) *in silico* analysis of dysregulated genes and proteins to identify their potential as prognostic and predictive markers in canine bladder cancer; and (3) selection of five previous studies with transcriptome data, extraction of common gene information from these studies and validation with The Cancer Genome Atlas (TCGA) data.

### Meta-Analysis

To identify previously published papers to include in our meta-analysis, we performed a literature search in PubMed, MEDLINE, and Scielo databases using the indexing terms “bladder” AND “carcinoma” AND “dog” with no restriction regarding the year of publication. Then, we reviewed the reference section of the selected manuscripts and performed a manual search in the most relevant journals with oncology backgrounds to ensure that we included the highest number of available manuscripts.

Next, we selected manuscripts by title and abstract, including scientific articles that evaluated genes or proteins in canine bladder carcinomas. In this step, we excluded review manuscripts, case reports, and retrospective studies including only survival analysis. Then, we analyzed each included manuscript and selected scientific papers that evaluated genes or proteins in canine bladder samples and compared them with their counterparts in normal bladder tissues. In this step, we excluded manuscripts using only cell lines, manuscripts that compared bladder carcinomas with cystitis as a control (with no normal sample comparison), and manuscripts evaluating only bladder carcinomas with no comparison to normal bladder tissue. Our first search was performed on December 12, 2019, and it was last updated on April 2, 2020.

From the selected manuscripts, we retrieved information regarding each dysregulated gene or protein, the “*p*-value” for each gene or protein (comparison between bladder cancer and normal bladder tissues), and survival data.

### *In silico* Analysis

The *in silico* analysis of each evaluated gene and protein was performed using free online tools. In the first step, we selected only the dysregulated or mutated genes and evaluated them with STRING (https://string-db.org/) to determine the proteins related to each gene of interest, using *Canis lupus familiaris* as a reference for comparisons. Then, we used only proteins for the subsequent analysis. We opted to evaluate only proteins in our *in silico* study due to the utility of proteins as prognostic and predictive markers.

The dysregulated proteins were together (upregulated and downregulated) and independently (upregulated or downregulated) using the online Search Tool for the Retrieval of Interacting Genes (STRING; https://string-db.org/) to generate protein–protein interaction (PPI) networks. We considered only STRING interactions of high confidence (0.700), and we hid the disconnected nodes for better visualization. The interactions considered to generate the PPI networks were coexpression, co-occurrence, database, and neighborhood interactions.

### Gene Ontology

Gene ontology (GO) analysis was performed to understand the biological role of proteins of interest among different species. The selected proteins were analyzed using Enrichr (https://amp.pharm.mssm.edu/Enrichr/). The analyzed information was retrieved from Enrichr and submitted to REVIGO (http://revigo.irb.hr/) to organize and visualize the enriched GO terms. We considered the three GO categories (biological process, cellular component, and molecular function) independently. However, since the biological process and molecular function terms have a higher chance of providing prognostic and predictive information, we focused on these two categories, and the enrichment analyses were performed using the same group of GO terms. For this analysis, we included only curated human annotations.

### Transcriptome Data Retrieved From the Previous Literature

We selected three previous studies that evaluated the transcriptome (RNA-seq) of canine bladder carcinoma, with the datasets available online via the NCBI short-read archive (SRA) under BioProject ID PRJNA559406 (18), GEO database (ref: GSE24152) (1), and DDBJ Sequenced Read Archive repository (http://trace.ddbj.nig.ac.jp/dra/index_e.html) with accession number DRA005844. In addition, we also included one manuscript evaluating the transcriptome of canine bladder cancer using microarray data (13) and one manuscript that performed both mRNA-seq and exome-seq (3). For both studies (3, 13), mRNA data were obtained from the Supplementary Materials.

The genes differentially expressed between TCC and normal bladder were selected with the following criteria: *p* < 0.05 and a fold change of 2.5 or higher in either direction. A Venn diagram was generated using an online tool (http://www.interactivenn.net/) (37). Furthermore, the common differentially expressed genes among the five studies were validated using 344 bladder carcinoma samples from the TCGA database.

### The Cancer Genome Atlas (TCGA) and The Cancer Proteome Atlas (TCPA) Cross-Validation

Due to the lack of a veterinary database with deposited information regarding the survival of canine patients with bladder carcinoma, we selected the most relevant proteins and validated them using 344 human samples from patients with muscle-invasive bladder carcinoma from TCGA (https://www.cancer.gov/tcga). The selected proteins were chosen based on the respective *p*-value and the biological function of the protein as previously described for other tumor subtypes. Then, the cross-validated proteins were evaluated via TCPA (https://tcpaportal.org/tcpa/index.html) (38). We considered proteins with a 5% interval of confidence or a *p*-value lower than 0.05.

In addition, we performed two different analyses using TCPA. First, we used the “visualization” tool to perform a global analysis to evaluate interactions among genes, including negative and positive interactions. In this way, we selected genes and pathways of human bladder cancer differentially implicated as possible markers to be used in canine bladder cancer. In the second analysis, we evaluated the overall survival of 344 human bladder cancer patients according to protein expression levels (high vs. low). In this analysis, we selected all genes in human bladder cancer with prognostic value. The Kaplan–Meier curves were generated using the TCPA online tool “individual cancer analysis” (https://tcpaportal.org/tcpa/analysis.html) (38, 39).

## Results

### Meta-Analysis

A total of 385 manuscripts were identified in the initial search. Then, according to the inclusion criteria, after reading the title and abstract, we excluded 329 manuscripts, and after reading the full manuscript, 25 of them met our inclusion criteria (Supplementary Figure 2). Next, we divided the selected manuscripts into two categories: manuscripts with global transcriptome analysis (*N* = 5) and manuscripts with reported isolated genes or proteins (*N* = 20). A complete list of the selected manuscripts can be found in Table 1.

**Table 1:** Manuscripts (*N* = 25) meeting the inclusion criteria and from published studies of canine bladder carcinoma.

**References**	**Manuscript title**	**Normal samples**	**Bladder cancer samples**	**Muscle invasion information**	**Gene or protein**	**Dysregulation**	***P*-value**
Maeda et al. (2)	Comprehensive gene expression analysis of canine invasive urothelial bladder carcinoma by RNA-Seq	*N* = 5	*N* = 11	Yes	Large-scale transcriptome analysis	No change	–
Tsuboi et al. (20)	Assessment of HER2 expression in canine urothelial carcinoma of the urinary bladder	*N* = 8	*N* = 23	No	HER2	Upregulated	*P* = 0.0288
Aupperle-Lellbach et al. (21)	Diagnostische aussagekraft der BRAF-mutation V595E in Urinproben, Ausstrichen, und Bioptaten Beim Kaninen ÜBergangszellkarzinom	*N* = 3	*N* = 43	No	BRAF V595E	Upregulated	N/R
Millanta et al. (22)	Overexpression of HER-2 via immunohistochemistry in canine urinary bladder transitional cell carcinoma—a marker of malignancy and possible therapeutic target	*N* = 5	*N* = 23	No	HER2	Upregulated	*P* < 0.05
Dhawan et al. (1)	Naturally occurring canine invasive urothelial carcinoma harbors luminal and basal transcriptional subtypes found in human muscle-invasive bladder cancer	*N* = 4	*N* = 29	No	Large-scale transcriptome analysis	No change	–
Walters et al. (23)	Expression of the receptor tyrosine kinase targets PDGFR-β, VEGFR2, and KIT in canine transitional cell carcinoma	*N* = 10	*N* = 30	No	PDGFR-β, VEGFR, c-KIT	Upregulated	*P* ≤ 0.001, *P* = 0.4268, *P* = 0.2453
Mohammed et al. (24)	Prostaglandin E2 concentrations in naturally occurring canine cancer	*N* = 10	*N* = 22	No	PGE2	Upregulated	*P* < 0.05
Dhawan et al. (25)	DNMT1: an emerging target in the treatment of invasive urinary bladder cancer	*N* = 6	*N* = 22	No	DNMT1	Upregulated	*P* < 0.02
Suárez-Bonnet et al. (26)	Expression of cell cycle regulators, 14-3-3σ and p53 proteins, and vimentin in canine transitional cell carcinoma of the urinary bladder	*N* = 5	*N* = 19	No	14-3-3σ, p53, vimentin	Upregulated, Upregulated, Downregulated	*P* = 0.0344, *P* = 0.044, *P* = 0.042
Yamazaki et al. (27)	SiRNA knockdown of the DEK nuclear protein mRNA enhances apoptosis and chemosensitivity of canine transitional cell carcinoma cells	*N* = 6	*N* = 14	No	DEK	Upregulated	*P* < 0.05
Dhawan et al. (25)	Targeting folate receptors to treat invasive urinary bladder cancer	*N* = 8	*N* = 74	Yes	FRs (folate receptors)	Upregulated	*P* < 0.0062
Clemo et al. (28)	Immunohistochemical evaluation of canine carcinomas with monoclonal antibody B72.3	*N* = 5	*N* = 13	No	TAG-72	Upregulated	N/R
Clemo et al. (29)	Immunoreactivity of canine transitional cell carcinoma of the urinary bladder with monoclonal antibodies to tumor-associated glycoprotein 72	*N* = 8	*N* = 51	No	TAG-72	Upregulated	*P* < 0.05
Hanazono et al. (30)	Immunohistochemical expression of p63, Ki67, and beta-catenin in canine transitional cell carcinoma and polypoid cystitis	*N* = 5	*N* = 25	No	p63, beta-catenin, Ki67	Downregulated, Downregulated, Upregulated	*P* < 0.01, *P* < 0.05, *P* < 0.01
Hanazono et al. (30)	Epidermal growth factor receptor expression in canine transitional cell carcinoma	*N* = 5	*N* = 25	No	EGFR	Upregulated	*P* < 0.01
Khan et al. (31)	Expression of cyclooxygenase-2 in transitional cell carcinoma of the urinary bladder in dogs	*N* = 8	*N* = 21	No	COX-2, COX-1	Upregulated, No change	N/R
Finotello et al. (32)	Lipoxygenase-5 expression in canine urinary bladder: normal urothelium, cystitis, and transitional cell carcinoma	*N* = 10	*N* = 29	No	LOX-5, COX-2	Upregulated	*P* < 0.01
Rankin et al. (33)	Identification of survivin, an inhibitor of apoptosis, in canine urinary bladder transitional cell carcinoma	*N* = 46	*N* = 41	No	Survivin	Upregulated	*P* < 0.001
Rankin et al. (33)	Comparison of distributions of survivin among tissues from urinary bladders of dogs with cystitis, transitional cell carcinoma, or histologically normal urinary bladders	*N* = 46	*N* = 41	No	Survivin	Upregulated	*P* = 0.07
Espinosa de Los Monteros et al. (34)	Coordinate expression of cytokeratins 7 and 20 in feline and canine carcinomas	*N* = 6	*N* = 14	No	CK7, CK 20	Upregulated	N/R
LeRoy et al. (35)	Canine prostate carcinomas express markers of urothelial and prostatic differentiation	*N* = 8	*N* = 19	No	CK7	Upregulated	N/R
Parker et al. (18)	RNAseq expression patterns of canine invasive urothelial carcinoma reveal two distinct tumor clusters and shared regions of dysregulation with human bladder tumors	*N* = 5	*N* = 15	No	Large-scale transcriptome analysis	No change	–
Sakai et al. (36)	ErbB2 copy number aberration in canine urothelial carcinoma detected by a digital polymerase chain reaction assay	*N* = 39	*N* = 36	No	ERBB2	Upregulated	*P* < 0.0001
Dhawan et al. (13)	Comparative gene expression analyses identify luminal and basal subtypes of canine invasive urothelial carcinoma that mimic patterns in human invasive bladder cancer	*N* = 4	*N* = 18	No	Large-scale transcriptome analysis	No change	–
Ramsey et al. (3)	Cross-species analysis of the canine and human bladder cancer transcriptome and exome.	*N* = 3	*N* = 6	No	Large-scale transcriptome analysis	No change	–

In the studies involving isolated protein analysis, HER-2 was the most studied protein (3/20), followed by TAG-72 (2/20), COX-2 (2/20), survivin (2/20), and CK7 (2/20). The remaining proteins were evaluated in only one previous study each (Table 1). In the protein–protein interaction analysis, we identified one interaction network with most interactions involving P53. After enrichment analysis using Enrichr, we evaluated the most common ontological processes associated with each previously published protein, and we identified several processes related to tyrosine kinase regulation, cell communication and signaling, and the MAPK pathway (Supplementary Figure 3 and Supplementary Table 1).

### *In silico* Analysis of Canine Transcriptome Data

**Figure 1:**
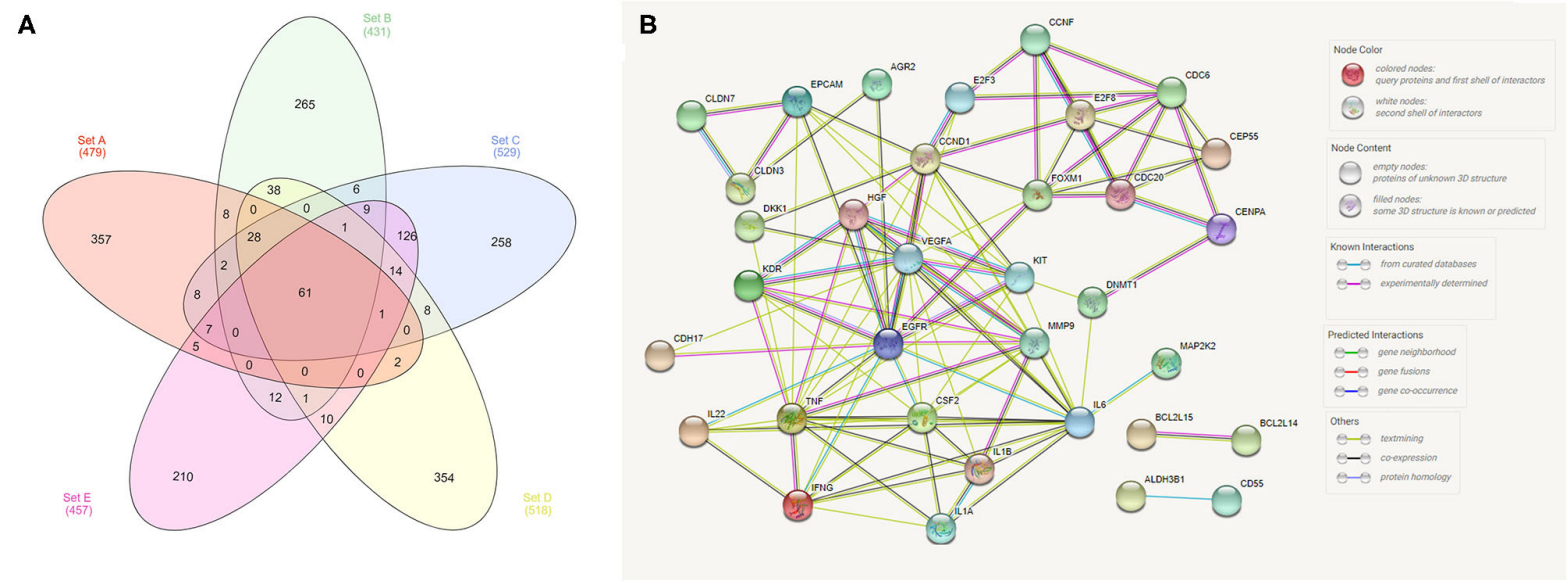
Analysis of transcriptome data from the five previously published studies in canine bladder transitional cell carcinoma. **(A)** Venn diagram demonstrating the number of commonly dysregulated genes (61) among the five studies. **(B)** Protein–protein interaction (PPI) network of the 61 dysregulated genes. Several interactions were seen among genes, including *EGFR* and *VEGFA*, with high degrees of interaction. The Venn diagram was generated online (http://www.interactivenn.net/) using the five available manuscripts with transcriptome data, and the PPI was generated with the 61 genes commonly dysregulated among the five studies using STRING (https://string-db.org/).

In our meta-analysis, we identified five previous studies containing transcriptome data, and in the most recent study (18), the authors cross-validated their findings with three other published manuscripts (1, 2, 13). Thus, we opted to analyze the transcriptome data from these five previous studies and cross-validate them with TCGA data. In our cross-validation analysis using the five previous veterinary studies, we identified 61 dysregulated genes (Figure 1), including CD55, IL17B, EGFR, CDH17, and CDH26. Moreover, we performed a PPI analysis among these genes and demonstrated a high interaction among them, with VEGFA, EGFR, TNF, and CCND1 being central genes in the interaction network (Figure 1).

We identified 35 dysregulated genes in the cross-validation analysis of the five veterinary studies and the TCGA data (Table 2).

**Table 2:** Dysregulated genes identified via cross-validation of data from the five veterinary studies with transcriptome data and data from 344 human samples from The Cancer Genome Atlas (TCGA).

**Gene/protein**	**Chromosome**	**Gene ID**	**Regulation**	**Function**
BRAF V595E	16	475526	Upregulated	Proto-oncogene, serine/threonine kinase
PTGER2	8	403797	Upregulated	Prostaglandin E receptor 2
ERBB2/HER-2	9	403883	Upregulated	Receptor tyrosine kinase 2
CHST4	5	489722	Upregulated	Carbohydrate sulfotransferase 4
PIGR	7	474357	Upregulated	Polymeric immunoglobulin receptor
S100A14	7	612322	Upregulated	Calcium binding protein A14
ADGRF1	12	474927	Upregulated	Adhesion G protein-coupled receptor F1
AGR2	14	482333	Upregulated	Anterior gradient 2, protein disulfide isomerase
Irgm 1	11	606863	Downregulated	Immunity-related GTPase family M protein-like
TP53	5	403869	Downregulated	Tumor protein
ZFP36	1	484510	Downregulated	Ring finger protein
E2F2	2	100855664	Upregulated	Transcription factor 2
IFI16	38	488622	Upregulated	Interferon-activatable protein 203
EGFR	18	404306	Upregulated	Epidermal growth factor receptor
ERK1/2	26	477575	Upregulated	Mitogen-activated protein kinase 1
NfkB-RelA	12	481711	Upregulated	NFKB inhibitor like 1
MAP2K3	5	489547	Upregulated	Mitogen-activated protein kinase kinase 3
IL1A	17	403782	Upregulated	Interleukin 1 alpha
RABL6	9	480675	Upregulated	RAS oncogene family like 6
CCND1	18	449028	Upregulated	Cyclin D1
FOXM1	27	486743	Upregulated	Forkhead box M1
E2F3	35	488239	Upregulated	Transcription factor 3
FOX01	6	609116	Upregulated	RNA-binding fox-1 homolog 1
IL6	14	403985	Upregulated	Interleukin 6
IL1B	17	403974	Upregulated	Interleukin 1 beta
CSF2	11	403923	Upregulated	Colony-stimulating factor 2
TNF	12	403922	Upregulated	Tumor necrosis factor
IFNG	10	403801	Upregulated	Interferon gamma
IL22	10	481153	Upregulated	Interleukin 22
HGF	18	403441	Upregulated	Hepatocyte growth factor
VEGF	12	403802	Upregulated	Vascular endothelial growth factor A
PDGFR-B	4	442985	Upregulated	Platelet-derived growth factor receptor beta
VEGFR2	13	482154	Upregulated	Kinase insert domain receptor
DNMT1	20	476715	Upregulated	DNA methyltransferase 1
SFN	2	487351	Upregulated	Stratifin

### Cross-Validation With Human Bladder Cancer

**Figure 2:**
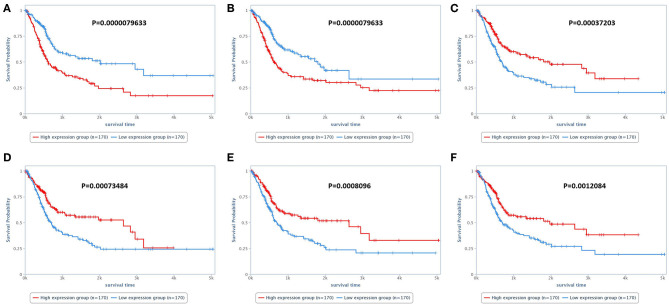
Survival analysis of human patients with bladder cancer. **(A)** Survival of patients according to Annexin 1 expression. Patients presenting high Annexin 1 expression experienced a shorter survival time than patients with low Annexin 1 expression. **(B)** Overall survival of patients according to TAZ expression. Patients with high TAZ expression experienced a shorter survival time than patients with low TAZ expression. **(C)** Overall survival according to SRC expression. Patients with lower SRC expression experienced a shorter survival time than patients with low SRC expression. **(D–F)** Overall survival according to SF2, ARID1A, and GATA3 expression, respectively. For these three proteins, patients with lower expression experienced a shorter survival time than those with higher expression. The survival analysis was performed using the TCPA online tool (https://tcpaportal.org/tcpa/survival_analysis.html).

In the analysis of the human bladder cancer samples, we identified several positive and negative protein correlations. In the PPI, many proteins from the serine/threonine and tyrosine kinase family, such as EGFR, ERK2, ERBB2, and BRAF, were observed. In addition, we identified 28 proteins with prognostic value in human bladder cancer (Table 3). Among these proteins, only two (EGFR and BRAF) were previously studied in canine bladder cancer (2/28). The top six proteins with prognostic value were Annexin 1, TAZ, SF2, SRC, ARID1A, and GATA3 (Figure 2).

**Table 3:** Proteins in human bladder cancer associated with overall survival in the cohort of 344 patients.

**Protein**	**Gene**	**Cox P**	**Log-rank P**
ANNEXIN1	ANXA1	0.000039282	0.0000079633
TAZ	TAZ	0.012031	0.00016332
SRC	SRC	0.00058508	0.00037203
SF2	SRSF1	0.033481	0.00073484
ARID1A	ARID1A	0.021321	0.0008096
GATA3	GATA3	0.0015211	0.0012084
BAK	BAK1	0.068011	0.0012196
EGFR	EGFR	0.006267	0.0016964
CD20	MS4A1	0.049073	0.0071387
SCD1	SCD1	0.10015	0.0078273
CABL	ABL	0.028914	0.011089
GATA6	GATA6	0.18358	0.014776
BAP1C4	BAP1	0.41571	0.015214
RICTOR	RICTOR	0.010638	0.015853
AXL	AXL	0.15225	0.015863
SMAD3	SMAD3	0.0048029	0.017136
BRAF_pS445	BRAF	0.068071	0.017645
SMAC	DIABLO	0.0039151	0.017688
BECLIN	BECN1	0.027124	0.018469
PARPCLEAVED	PARP1	0.21592	0.018488
ADAR1	ADAR	0.050259	0.022731
SHC_pY317	SHC1	0.23036	0.027714
TRANSGLUTAMINASE	TGM2	0.53787	0.036741
ANNEXINVII	ANXA7	0.36004	0.045156
PEA15	PEA15	0.15168	0.046395
PKCALPHA	PRKCA	0.056178	0.047571
MEK1	MAP2K1	0.65034	0.051509
CAVEOLIN1	CAV1	0.03709	0.058778

## Discussion

Human bladder cancer molecular findings are widely described in the literature, making it possible to reanalyze these data to provide new insights for comparative oncology. Although dogs can be considered models of human bladder cancer, few studies have provided a full description of canine bladder cancer molecular data. Since most canine bladder cancer studies have published isolated assessments of different proteins, the present study extracted these data and evaluated them together to understand how these proteins interact with each other. However, it is important to consider the limitations of each study, including differences in terms of tumor stage and therapeutic protocols used in each publication. One limitation of our study is the use of online tools such as Enrichr that use Fisher's exact test, which is statistically more likely to identify larger pathways than smaller pathways as significant. Thus, we selected the upper size limit for the size of gene sets to avoid misinterpretation.

In our meta-analysis, after the first search, several studies were excluded because they had no matched normal tissue analysis (*N* = 31/56). The inclusion of normal tissue is important to establish a pattern of expression between normal and cancer tissue. During the carcinogenic process, cancer cells can change their expression profile with gains or losses of expression of several genes, and it is important to include normal samples to avoid bias (40). In addition, for some *in silico* analyses, it is necessary to have a *p*-value related to the protein expression in tumor compared to normal tissues.

The meta-analysis demonstrated that most of the veterinary studies did not evaluate muscle invasion (23/25) by the tumor or did not provide clear information regarding this topic. Thus, as a future direction for canine studies evaluating transitional cell carcinoma from the bladder, we strongly suggest that the authors evaluate muscle invasion to provide stronger evidence regarding dogs being models of human bladder cancer. In addition, this lack of information about muscle invasiveness may have introduced some confounding factors that influenced the results. Additionally, it is difficult to make cross-species validations and assumptions given the lack of information in veterinary studies.

Most of the published manuscripts that met the inclusion criteria evaluated one to three proteins or genes, and only five previous manuscripts performed large-scale analyses on canine bladder carcinomas. These large-scale transcriptome studies used RNA-seq technology of the tumor samples. On the other hand, new single-cell sequencing technologies have been used in recent years to provide more specific information regarding the transcriptome of human cancers (41). However, to the best of our knowledge, there is no information regarding single-cell sequencing in canine bladder cancer. The single-cell technology allows the evaluation of tumor transcriptome excluding other cell-type, such as stromal, endothelial, and inflammatory cells.

From the selected studies, we extracted protein or gene information from the manuscripts studying isolated proteins and evaluated these proteins together. All studies that reported isolated proteins were focused on the evaluation of oncogenes. Interestingly, most of them evaluated tyrosine kinase receptors, such as ERBB2, EGFR, VEGFR, and PDGFR (20, 23, 42). Our PPI analysis revealed a high number of interactions among these proteins, even though they were evaluated separately in each study. As such, future studies may benefit from our PPI analysis to evaluate the prognostic or predictive value of the identified proteins in canine bladder cancer. Interestingly, the ontology analysis of the studies with isolated proteins revealed several terms related to tyrosine kinase activity, phosphorylation, and alteration of the ERK1 and ERK2 cascade. Thus, previous studies have focused on the search for small-molecule inhibitor targets. However, no small-molecule inhibitors have yet been successfully proposed in the treatment of canine bladder cancer. Thus, tyrosine kinase receptors could be overrepresented in our analysis, and this result should be interpreted with caution.

The cross-validation of canine transcriptome data with TCGA data revealed 35 genes with a high probability of presenting dysregulation in both human and canine bladder cancer. Since these data were obtained from five different canine studies and 344 human samples from TCGA, they are promising and could be used for further investigation. Though it has been difficult to identify potential markers to be tested in future studies, our list provides markers with strong potential that are upregulated or downregulated in both human and canine bladder cancer. One important limitation of our analysis was the absence of muscle invasion information/standardization in canine samples. Thus, we can lack data regarding important genes related to muscle invasiveness, which is known as a poor prognostic finding. Nevertheless, choosing a gene from our list for future studies could be more promising than a random search. In addition, we analyzed the data from 344 human bladder cancer patients with muscle-invasive patterns to identify genes related to overall survival. Since survival data are usually absent in published studies in veterinary medicine, human survival data represents a unique opportunity to identify candidates related to prognosis in veterinary oncology.

Among the 28 proteins with prognostic value, we identified Annexin 1, GATA-3, and EGFR. Annexin 1 overexpression was previously associated with tumor progression and was considered an independent marker for metastasis-free survival (43). In addition, Annexin 1 expression was also associated with chemotherapy relapse and resistance in human bladder cancer (44). In the present meta-analysis, studies evaluating Annexin 1 expression in canine bladder tumors were not found. GATA3 is widely used in human medicine as a diagnostic marker (45, 46). Interestingly, in addition to its use as a diagnostic marker, GATA3 has been shown to be an important prognostic marker in human bladder cancer (45). Decreased GATA3 expression is associated with low recurrence-free survival, a high frequency of muscle invasiveness, and a tumor progression. In veterinary medicine, one previous review mentioned GATA3 expression in canine bladder cancer and showed GATA3 expression in a sample of canine bladder carcinoma (10). However, since it was a review, these authors did not evaluate canine bladder carcinoma samples. The corresponding author was contacted and kindly provided information regarding the GATA3 antibody used for the data. Overall GATA3 has the potential to be a prognostic marker for canine bladder cancer.

EGFR is an important marker in human bladder cancer and is associated with overall survival, muscle invasiveness, and tumor recurrence (47). Thus, EGFR overexpression has been studied and is a target for anti-EGFR therapies (48). In dogs, EGFR has previously been evaluated in canine bladder cancer (42). However, the authors evaluated the EGFR gene and protein in samples but provided no association with clinicopathological findings. Regardless, based on meta-analyses and *in silico* analyses, EGFR shows promising potential in terms of both prognostic and predictive value in canine bladder cancer. (49) evaluated an anti-EGFR monoclonal antibody in canine transitional carcinoma cells from the bladder *in vitro* and *in vivo*. The authors' findings suggested that this anti-EGFR monoclonal antibody could be promising for the treatment of dogs with bladder cancer. Thus, both humans and dogs can benefit from clinical trials involving anti-EGFR antibodies in dogs.

## Conclusion

The canine literature on bladder cancer has been focused on the evaluation of isolated markers with no association with patient survival. In addition, the lack of information regarding tumor muscle invasion can be considered an important limitation when comparing human and canine bladder tumors. Our *in silico* analysis involving canine and human transcriptome data provided several genes with the potential to be markers for both human and canine bladder tumors, and these genes should be considered for future studies on canine bladder cancer.

## Data Availability Statement

The original contributions presented in the study are included in the article/Supplementary Materials, further inquiries can be directed to the corresponding author.

## Author Contributions

VV and CF-A wrote the first manuscript draft. VV performed the meta-analysis and *in silico* analysis of the selected data. CF-A and RF checked the meta-analysis and the *in silico* data independently. RL-A and VG contributed constructive comments. CF-A supervised the project. All authors read and approved the final manuscript.

## Conflict of Interest

The authors declare that the research was conducted in the absence of any commercial or financial relationships that could be construed as a potential conflict of interest.

## References

[1] Dhawan D Hahn NM Ramos-Vara JÁ Knapp DW Naturally-occurring canine invasive urothelial carcinoma harbors luminal and basal transcriptional subtypes found in human muscle invasive bladder cancer. PLoS Genet. (2018) 14:e1007571 10.1371/journal.pgen.1007571 30089113 PMC6101404

[2] Maeda S Tomiyasu H Tsuboi M Inoue A Ishihara G Uchikai T Comprehensive gene expression analysis of canine invasive urothelial bladder carcinoma by RNA-Seq. BMC Cancer. (2018) 18:472 10.1186/s12885-018-4409-3 29699519 PMC5921755

[3] Ramsey SA Xu T Goodall C Rhodes AC Kashyap A He J Cross-species analysis of the canine and human bladder cancer transcriptome and exome. Genes Chromosomes Cancer. (2017) 56:328–43. 10.1002/gcc.22441 28052524

[4] Siegel RL Miller KD Jemal A Cancer statistics, 2020. CA Cancer J Clin. (2020) 70:7–30. 10.3322/caac.21590 31912902

[5] Bray F Ferlay J Soerjomataram I Siegel RL Torre LA Jemal A. Global cancer statistics 2018: GLOBOCAN estimates of incidence and mortality worldwide for 36 cancers in 185 countries. CA Cancer J Clin. (2018) 68:394–424. 10.3322/caac.21492 30207593

[6] Patrick D Fitzgerald S Sesterhenn I Davis C Kiupel M. Classification of Canine Urinary Bladder Urothelial Tumours Based on the World Health Organization/ International Society of Urological Pathology Consensus Classification. J Comp Path. (2006) 135:190–9. 10.1016/j.jcpa.2006.07.002 17054974

[7] Miyazaki J Nishiyama H. Epidemiology of urothelial carcinoma. Int J Urol. (2017) 24:730–4. 10.1111/iju.13376 28543959

[8] Cha JD Lourenço DB Korkes F. Analysis of the association between bladder carcinoma and arsenic concentration in soil and water in southeast Brazil. Int Braz J Urol. (2018) 44:906–13. 10.1590/s1677-5538.ibju.2017.0543 30044600 PMC6237532

[9] Glickman LT Schofer FS McKee LJ Reif JS Goldschmidt MH. Epidemiologic study of insecticide exposures, obesity, and risk of bladder cancer in household dogs. J Toxicol Environ Health. (1989) 28:407–14. 10.1080/15287398909531360 2593174

[10] Knapp DW Ramos-Vara JA Moore GE Dhawan D Bonney PL Young KE. Urinary bladder cancer in dogs, a naturally occurring model for cancer biology and drug development. ILAR, J. (2014) 55:100–18. 10.1093/ilar/ilu018 24936033

[11] Grzegółkowski P Kaczmarek K Lemiński A Soczawa M Gołab A Słojewski M. Assessment of the infiltrative character of bladder cancer at the time of transurethral resection: a single center study. Cent European J Urol. (2017) 70:22–6. 28461983 10.5173/ceju.2017.768 PMC5407325

[12] Hernández-Fernández C Herranz-Amo F Moralejo-Gárate M Subirá-Ríos D Caño-Velasco J Barbas-Bernardos G. Infiltrating bladder cancer: prognostic factors, follow-up and treatment of relapses. Actas Urol Esp. (2017) 41:352–8. 10.1016/j.acuroe.2016.07.010 27561847

[13] Dhawan D Paoloni M Shukradas S. Comparative Gene Expression Analyses Identify Luminal and Basal Subtypes of Canine Invasive Urothelial Carcinoma That Mimic Patterns in Human Invasive Bladder Cancer. PLoS One. (2015) 10:e0136688. 10.1371/journal.pone.0136688 26352142 PMC4564191

[14] Jalanko T de Jong J Gibb EA Seiler R Black P Genomic Subtyping in Bladder Cancer. Curr Urol Rep. (2020) 21:9 10.1007/s11934-020-0960-y 32166460

[15] Inamura K. Bladder Cancer: New Insights into Its Molecular Pathology. Cancers (Basel). (2018) 10:E100. 10.3390/cancers10040100 29614760 PMC5923355

[16] Cancer Genome Atlas Research Network (2014). Comprehensive molecular characterization of urothelial bladder carcinoma. Nature 507, 315–322. 10.1038/nature12965 24476821 PMC3962515

[17] Knapp DW Dhawan D Ramos-Vara JA Ratliff TL Cresswell GM Utturkar S. Naturally-Occurring Invasive Urothelial Carcinoma in Dogs, a Unique Model to Drive Advances in Managing Muscle Invasive Bladder Cancer in Humans. Front Oncol. (2020) 9:1493. 10.3389/fonc.2019.01493 32039002 PMC6985458

[18] Parker HG Dhawan D Harris AC. RNAseq expression patterns of canine invasive urothelial carcinoma reveal two distinct tumor clusters and shared regions of dysregulation with human bladder tumors. BMC Cancer. (2020) 20:251. 10.1186/s12885-020-06737-0 32209086 PMC7092566

[19] Mochizuki H Shapiro SG Breen M. Detection of BRAF Mutation in Urine DNA as a Molecular Diagnostic for Canine Urothelial and Prostatic Carcinoma. PLoS One. (2015) 10:e0144170. 10.1371/journal.pone.0144170 26649430 PMC4674145

[20] Tsuboi M Sakai K Maeda S Chambers JK Yonezawa T Matsuki N. Assessment of HER2 expression in canine urothelial carcinoma of the urinary bladder. Vet Pathol. (2019) 56:369–76. 10.1177/0300985818817024 30612533

[21] Aupperle-Lellbach H Grassinger J Hohloch C Kehl A Pantke P. Diagnostische aussagekraft der BRAF-mutation V595E in urinproben, ausstrichen und bioptaten beim kaninen Übergangszellkarzinom [Diagnostic value of the BRAF variant V595E in urine samples, smears and biopsies from canine transitional cell carcinoma]. Tierarztl Prax Ausg K Kleintiere Heimtiere. (2018) 46:289–95. 10.15654/TPK-180554 30541168

[22] Millanta F Impellizeri J mcsherry L Rocchigiani G Aurisicchio L Lubas G. Overexpression of HER-2 via immunohistochemistry in canine urinary bladder transitional cell carcinoma - a marker of malignancy and possible therapeutic target. Vet Comp Oncol. (2018) 16:297–300. 10.1111/vco.12345 28871659

[23] Walters L Martin O Price J Sula MM. Expression of receptor tyrosine kinase targets PDGFR-β, VEGFR2 and KIT in canine transitional cell carcinoma. Vet Comp Oncol. (2018) 16:E117–22. 10.1111/vco.12344 28884928

[24] Mohammed SI Coffman K Glickman NW Hayek MG Waters DJ Schlittler D. Prostaglandin E2 concentrations in naturally occurring canine cancer. Prostaglandins Leukot Essent Fatty Acids. (2001) 64:1–4. 10.1054/plef.2000.0231 11161579

[25] Dhawan D Ramos-Vara J Hahn N Waddell J Olbricht G Zheng R. DNMT1: an emerging target in the treatment of invasive urinary bladder cancer. Urol Oncol. (2013) 31:1761–9. 10.1016/j.urolonc.2012.03.015 22609058

[26] Suárez-Bonnet A Herráez P Aguirre M Suárez-Bonnet E Andrada M Rodríguez F. Expression of cell cycle regulators, 14-3-3σ and p53 proteins, and vimentin in canine transitional cell carcinoma of the urinary bladder. Urol Oncol. (2015) 33:332.e1–7. 10.1016/j.urolonc.2015.04.006 25979650

[27] Yamazaki H Iwano T Otsuka S Kagawa Y Hoshino Y Hosoya K. Sirna knockdown of the DEK nuclear protein mrna enhances apoptosis and chemosensitivity of canine transitional cell carcinoma cells. Vet J. (2015) 204:60–5. 10.1016/j.tvjl.2015.02.009 25773167

[28] Clemo FA denicola DB Delaney LJ. Immunohistochemical evaluation of canine carcinomas with monoclonal antibody B72.3. Vet Pathol. (1993) 30:140–5. 10.1177/030098589303000206 8470336

[29] Clemo FA denicola DB Carlton WW Walker E Morrison WB. Immunoreactivity of canine transitional cell carcinoma of the urinary bladder with monoclonal antibodies to tumor-associated glycoprotein 72. Vet Pathol. (1995) 32:155–61. 10.1177/030098589503200209 7771056

[30] Hanazono K Nishimori T Fukumoto S Kawamura Y Endo Y Kadosawa T. Immunohistochemical expression of p63, Ki67 and β-catenin in canine transitional cell carcinoma and polypoid cystitis of the urinary bladder. Vet Comp Oncol. (2016) 14:263–9. 10.1111/vco.12095 24758385

[31] Khan KN Knapp DW Denicola DB Harris RK. Expression of cyclooxygenase-2 in transitional cell carcinoma of the urinary bladder in dogs. Am J Vet Res. (2000) 61:478–81. 10.2460/ajvr.2000.61.478 10803639

[32] Finotello R Schiavo L Ressel L Frohmader A Silvestrini P Verin R. Lipoxygenase-5 expression in canine urinary bladder: normal urothelium, cystitis and transitional cell carcinoma. J Comp Pathol. (2019) 170:1–9. 10.1016/j.jcpa.2019.05.001 31375151

[33] Rankin WV Henry CJ Turnquist SE Turk JR Beissenherz ME Tyler JW. Identification of survivin, an inhibitor of apoptosis, in canine urinary bladder transitional cell carcinoma. Vet Comp Oncol. (2008) 6:141–50. 10.1111/j.1476-5829.2007.00150.x 19178674

[34] Espinosa de los Monteros A Fernández A Millán MY Rodríguez F Herráez P Martín de las Mulas J. Coordinate expression of cytokeratins 7 and 20 in feline and canine carcinomas. Vet Pathol. (1999) 36:179–90. 10.1354/vp.36-3-179 10332826

[35] leroy BE Nadella MV Toribio RE Leav I Rosol TJ. Canine prostate carcinomas express markers of urothelial and prostatic differentiation. Vet Pathol. (2004) 41:131–40. 10.1354/vp.41-2-131 15017026

[36] Sakai K Maeda S Saeki K Yoshitake R Goto-Koshino Y Nakagawa T. Erbb2 copy number aberration in canine urothelial carcinoma detected by a digital polymerase chain reaction assay. Vet Pathol. (2020) 57:56-65. 10.1177/0300985819879445 31640537

[37] Heberle H Meirelles GV da Silva FR Telles GP Minghim R. InteractiVenn: a web-based tool for the analysis of sets through Venn diagrams. BMC Bioinformatics. (2015) 16:169. 10.1186/s12859-015-0611-3 25994840 PMC4455604

[38] Li J Lu Y Akbani R Ju Z Roebuck P Liu W. TCPA: a resource for cancer functional proteomics data. Nature Methods. (2013) 10:1046–7. 10.1038/nmeth.2650 24037243 PMC4076789

[39] Li J Akbani R Zhao W Lu Y Weinstein J Mills G Liang H. Explore, visualize, and analyze functional cancer proteomic data using the cancer. Proteome Atlas Cancer Res. (2017) 77:e51–4. 10.1158/0008-5472.CAN-17-0369 29092939 PMC5679242

[40] Kosti I Jain N Aran D Butte A Sirota M. Cross-tissue Analysis of Gene and Protein Expression in Normal and Cancer Tissues. Sci Rep. (2016) 6:24799. 10.1038/srep24799 27142790 PMC4855174

[41] Cho B Yoon SH Lee D. Single-cell transcriptome maps of myeloid blood cell lineages in Drosophila. Nat Commun. (2020) 11:4483. 10.1038/s41467-020-18135-y 32900993 PMC7479620

[42] Hanazono K Fukumoto S Kawamura Y. Epidermal growth factor receptor expression in canine transitional cell carcinoma. J Vet Med Sci. (2015) 77:1–6. 10.1292/jvms.14-0032 25223345 PMC4349531

[43] Li C Shen K Huang L Huang H Wang Y Wu TF. Annexin-I overexpression is associated with tumour progression and independently predicts inferior disease-specific and metastasis-free survival in urinary bladder urothelial carcinoma. Pathology. (2010) 42:43–9. 10.3109/00313020903434405 20025479

[44] Yu S Meng Q Hu H Zhang M. Correlation of ANXA1 expression with drug resistance and relapse in bladder cancer. Int J Clin Exp Pathol. (2014) 7:5538–48. 25337195 PMC4203166

[45] Inoue S Mizushima T Fujita K Meliti A Ide H Yamaguchi S. GATA3 immunohistochemistry in urothelial carcinoma of the upper urinary tract as a urothelial marker and a prognosticator. Hum Pathol. (2017) 64:83–90. 10.1016/j.humpath.2017.04.003 28428106

[46] Agarwal H Babu S Rana C Kumar M Singhai A Shankhwar S. Diagnostic utility of GATA3 immunohistochemical expression in urothelial carcinoma. Indian J Pathol Microbiol. (2019) 62:244–50. 10.4103/IJPM.IJPM_228_18 30971548

[47] Hashmi A Hussain Z Irfan M Khan E Faridi N Naqvi H. Prognostic significance of epidermal growth factor receptor (EGFR) over expression in urothelial carcinoma of urinary bladder. BMC Urol. (2018) 18:59. 10.1186/s12894-018-0373-0 29879970 PMC5992678

[48] Karyagina T Ulasov A Slastnikova T Rosenkranz A Lupanova T Khramtsov Y. Targeted Delivery of (111)In Into the Nuclei of EGFR Overexpressing Cells via Modular Nanotransporters With Anti-EGFR Affibody. Front Pharmacol. (2020) 11:176. 10.3389/fphar.2020.00176 32194412 PMC7064642

[49] Nagaya T Okuyama S Ogata F Maruoka Y Knapp DW Karagiannis SN. Near infrared photoimmunotherapy targeting bladder cancer with a canine anti-epidermal growth factor receptor (EGFR) antibody. Oncotarget. (2018) 9:19026–38. 10.18632/oncotarget.24876 29721181 PMC5922375

