# Supplementary material for: A Comparative Meta-Analysis and in silico Analysis of Differentially Expressed Genes and Proteins in Canine and Human Bladder Cancer
Source: Front Vet Sci. 2020 Nov 16;7:558978. doi: 10.3389/fvets.2020.558978 (PMC7701042; doi:10.3389/fvets.2020.558978)
Supplement: Supplementary file 1 [file Data_Sheet_1.ZIP › Supplementary Figure 1.docx]

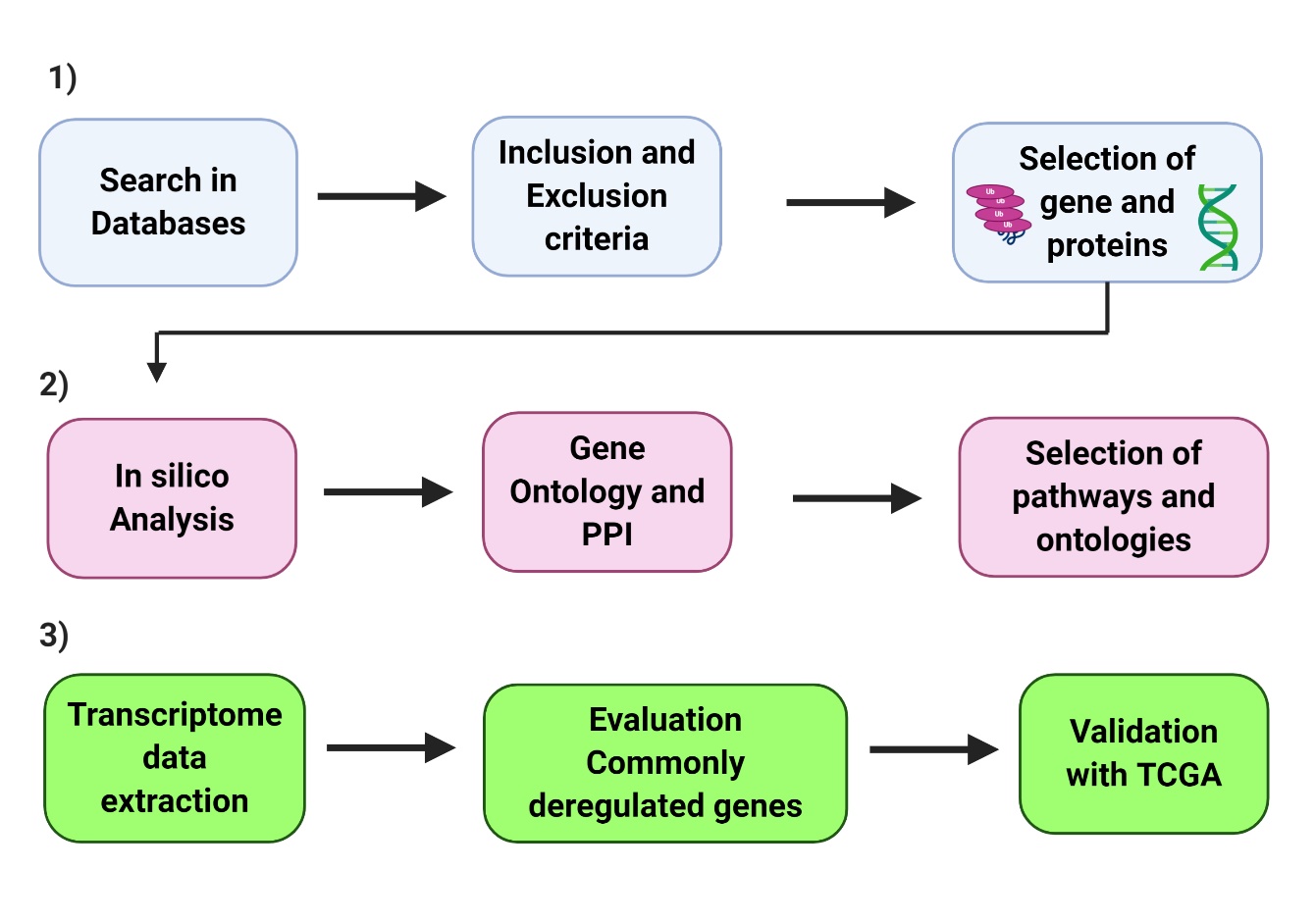
**Supplementary Figure 1**. Schematic representation of the manuscript design. 1) We searched different databases for manuscripts evaluating proteins or genes in canine bladder carcinoma. To the identified manuscripts, we applied different inclusion and exclusion criteria, and we extracted gene and protein information from the identified manuscripts. Next, we performed two different analyses based on the manuscript data. 2) Manuscripts with information regarding isolated genes or proteins were evaluated together. We extracted the name of the gene or protein and the p value to perform ontology and protein-protein interaction analyses. At the end of these analyses, we identified different pathways and ontological processes related to previously published data. 3) The manuscripts with large-scale transcriptome data were evaluated together in a separate analysis. We identified genes commonly dysregulated among studies and cross-validated these data with data from The Cancer Genome Atlas (TCGA). The diagram was generated using BioRender (<https://app.biorender.com/>).
