# Supplementary material for: A Comparative Meta-Analysis and in silico Analysis of Differentially Expressed Genes and Proteins in Canine and Human Bladder Cancer
Source: Front Vet Sci. 2020 Nov 16;7:558978. doi: 10.3389/fvets.2020.558978 (PMC7701042; doi:10.3389/fvets.2020.558978)
Supplement: Supplementary file 1 [file Data_Sheet_1.ZIP › Supplementary Figure 2.docx]

**Supplementary Figure 2**. Flowchart of the evaluated manuscripts. In the first search, 385 manuscripts were identified, and after applying all inclusion and exclusion criteria, we identified five manuscripts with large-scale transcriptome data and 20 manuscripts with isolated gene or protein data. The diagram was generated using BioRender (<https://app.biorender.com/>).


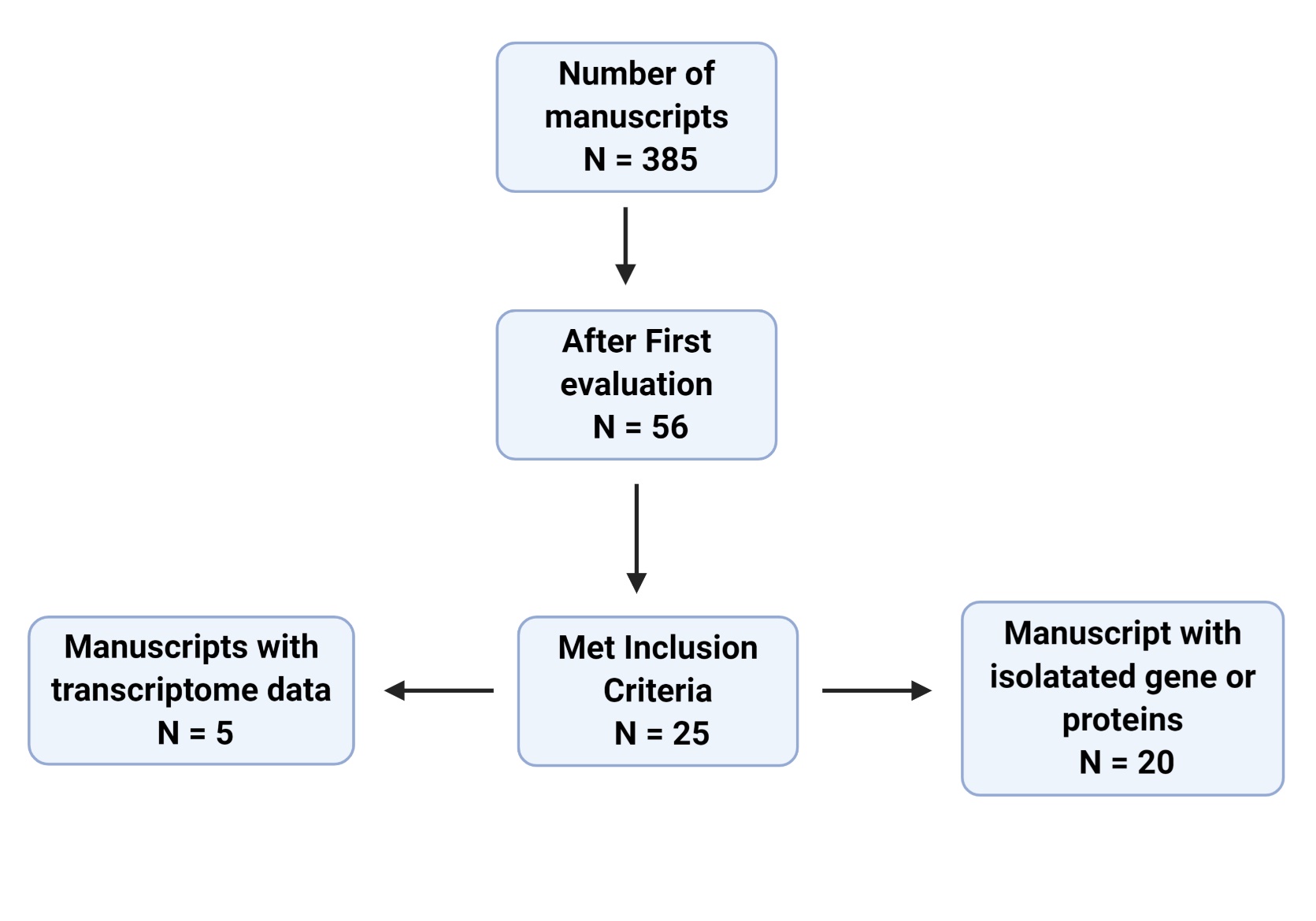


.
