# Supplementary material for: A Comparative Meta-Analysis and in silico Analysis of Differentially Expressed Genes and Proteins in Canine and Human Bladder Cancer
Source: Front Vet Sci. 2020 Nov 16;7:558978. doi: 10.3389/fvets.2020.558978 (PMC7701042; doi:10.3389/fvets.2020.558978)

**Supplementary Figure 3**. Ontology analysis of the isolated proteins extracted from previously published papers. A, B and C represent the visualized ontology data from REVIGO (<http://revigo.irb.hr/>), considering those ontological processes with a statistical difference e and excluding redundancies. D, E and F represent the data from the ontology analysis via Enrichr (<https://amp.pharm.mssm.edu/Enrichr/>), demonstrating the importance of a given ontological process with p-value (red bars). A and D are molecular function-related ontological processes, B and E are ontological processes related to cellular components, and C and F are biological process-related terms. This analysis revealed that the published literature has been focused on the study of oncogenes with tyrosine kinase properties. Regulation of tyrosine kinase receptors and phosphorylation were the most common terms.

.


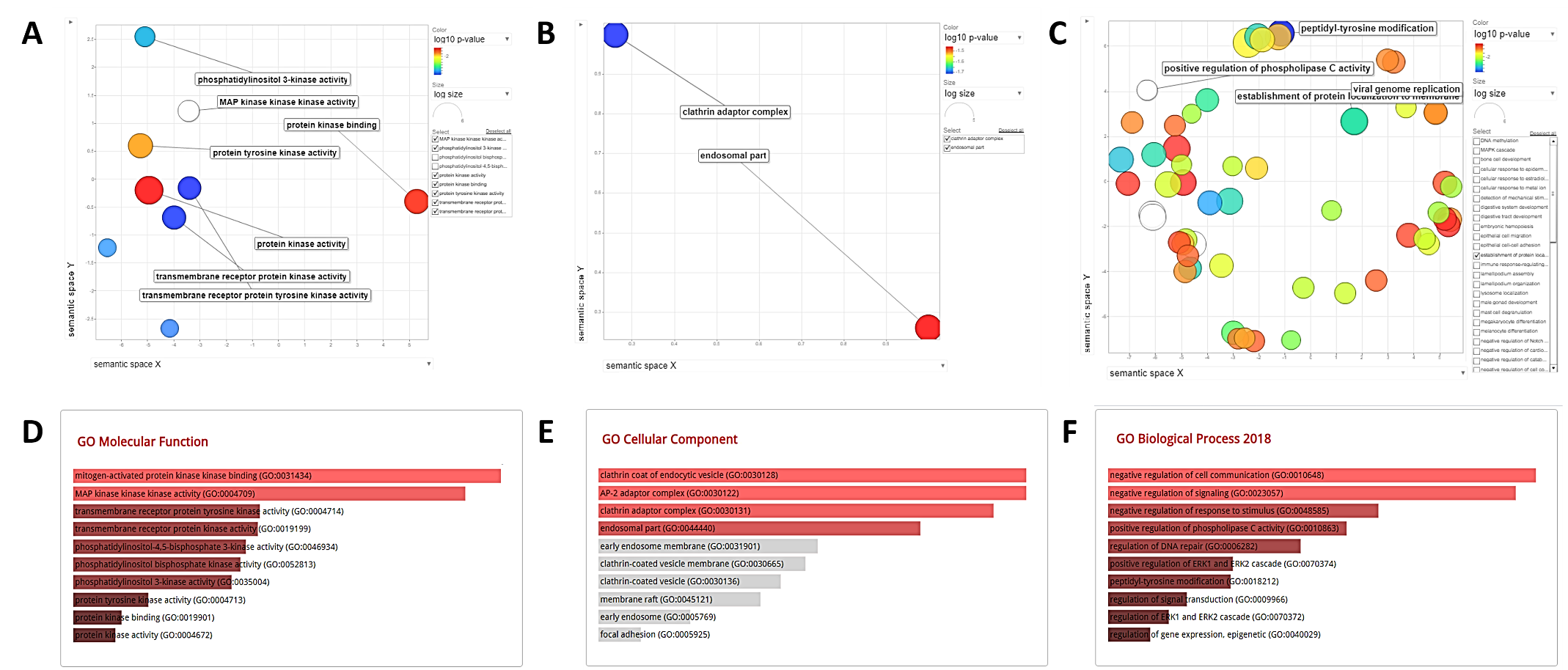

Supplement: Supplementary file 1 [file Data_Sheet_1.ZIP › Supplementary Figure 3.docx]
